# Complete Genome Sequence of *Mycoplasma pneumoniae* Type 2 Reference Strain FH Using Single-Molecule Real-Time Sequencing Technology

**DOI:** 10.1128/genomeA.01629-16

**Published:** 2017-02-23

**Authors:** Heta P. Desai, Shatavia S. Morrison, Maureen H. Diaz, Alvaro J. Benitez, Bernard J. Wolff, Jonas M. Winchell

**Affiliations:** Division of Bacterial Diseases, National Center for Immunization and Respiratory Diseases, Centers for Disease Control and Prevention, Atlanta, Georgia, USA

## Abstract

*Mycoplasma pneumoniae* type 2 strain FH was previously sequenced with Illumina (FH-Illumina) and 454 (FH-454) technologies according to Xiao et al. (2015) and Krishnakumar et al. (2010). Comparative analyses revealed differences in genomic content between these sequences, including a 6-kb region absent from the FH-454 submission. Here, we present a complete genome sequence of FH sequenced with the Pacific Biosciences RSII platform.

## GENOME ANNOUNCEMENT

*Mycoplasma pneumoniae* is a common cause of community-acquired pneumonia worldwide (1). Two primary strain types, type 1 and type 2, are recognized based on variations within the genes encoding the P1 adhesion molecule. Complete genomes of the reference strains M129 (type 1) and FH (type 2) have been available since 1996 and 2010, respectively (2–5). Comparative genomics relies on complete, curated references, as even minor differences may impact transmissibility or virulence. Single-molecule real-time (SMRT) sequencing generates long reads for assembly of genomes with improved quality, particularly across difficult-to-sequence regions. Here, we present a complete sequence of strain FH (FH-PacBio) using SMRT technology.

Genomic DNA was extracted with the MasterPure Complete DNA/RNA purification kit (Epicentre, Madison, WI, USA), and a 10-kb library was prepared for PacBio sequencing (Pacific Biosciences, Menlo Park, CA, USA). A paired-end Illumina sequencing library was also prepared using the NEBNext Ultra DNA kit (New England Biolabs, Ipswich, MA, USA). A total of 38,894 PacBio sequencing reads were assembled using the Hierarchical Genome Assembly Process (HGAP version 3) into a completely closed genome. Nucleotide accuracy was >99.9%, as indicated by the mapping of Illumina reads (*n* = 2,729,324) to the assembled genome. The genome size was 817,276 bp. Prokka version 1.8 was used to predict 757 coding sequences, 36 tRNAs, and one tmRNA (6). In a pairwise comparison, the FH-Illumina sequence (NZ_CP010546.1) was 69 bp shorter than FH-PacBio due to differences in a short tandem repeat region, and had only one fewer gene (7, 8). The largest discrepancy was found in comparing FH-454 (NC_017504.1) and FH-PacBio; a 6-kb region containing lipoprotein-encoding genes was absent in FH-454, confirming a previous observation in comparing FH-Illumina and FH-454 (9). This difference may be due to misassembly resulting from a neighboring repetitive element or a gene knockout experiment performed on the isolate prior to sequencing (2). Prior to the availability of the FH-Illumina and FH-PacBio sequences, this 6-kb difference was thought to be a differentiating feature between variants of type 2 *M. pneumoniae* isolates. However, it is now recognized as a common feature to all type 2 strains. FH-PacBio and FH-Illumina, which differed only by 24 single nucleotide polymorphisms, are more accurate reference genomes for FH and serve as superior representatives of type 2 *M. pneumoniae* genomes.

This analysis demonstrates the impact of sequencing technology and assembly methods on the generation of completely characterized reference genomes, which are necessary for understanding strain relatedness and identifying relevant features that may impact disease. The availability of well-curated genomes is imperative as whole-genome sequencing technology expands in public health and clinical laboratories.

### Accession number(s).

This whole-genome sequence has been deposited at GenBank under accession number CP017327 and at the Sequence Read Archive under accession number SRR3924617.
